# Community participation of patients 12 months post-stroke in Johannesburg, South Africa

**DOI:** 10.4102/phcfm.v5i1.426

**Published:** 2013-01-24

**Authors:** Witness Mudzi, Aimee Stewart, Eustasius Musenge

**Affiliations:** 1 Department of Physiotherapy, University of the Witwatersrand, South Africa; 2 Biostatistics and Epidemiology Division, University of the Witwatersrand, South Africa

## Abstract

**Background:**

Improvement in health-related quality of life (HRQL) is the main goal of rehabilitation. The ability of the stroke-patient to participate in various situations signifies successful rehabilitation. The aim of the study was to establish the extent of community participation and the barriers and facilitators to the participation for stroke patients after their discharge.

**Method:**

This study formed part of a larger study focusing on the impact of caregiver education on stroke survivors and their careers. This was a longitudinal study comprising 200 patients with first-time ischaemic stroke. Although the patients were followed up at home at 3 months, 6 months and 12 months post-stroke, this paper focuses on the 12-months follow-up participation results. Patient functional ability was measured by using the Barthel Index (BI) and the Rivermead Mobility Index (RMI), whereas participation was measured by using the International Classification of Functioning, Disability and Health (ICF) checklist. Descriptive statistics were used to analyse the data.

**Results:**

Patients experienced severe to complete difficulty when undertaking single and multiple tasks without help 12-months post-discharge. They struggled with the preparation of meals, household work and interpersonal interactions, and they had difficulties with community life and partaking in recreation and leisure activities. Immediate family and societal attitudes were viewed as facilitators to community participation whereas friends, transportation services and social security services were viewed as barriers to community participation.

**Conclusion:**

The patient-ability to socialise and participate in community issues is currently poor. The identified barriers to community participation need to be addressed in order to improve patient-participation in the community post-stroke.

## Abstrait

**Participation communautaire de patients 12 mois après avoir souffert d'un accident vasculaire cérébral à Johannesburg, en Afrique du Sud**

**Contexte:**

L'amélioration de la qualité de vie liée à la santé (QVLS) est le but principal de la réhabilitation. La capacité des patients ayant souffert d**’**accident vasculaire cérébral à participer à différentes situations montre que la réhabilitation est un succès. Le but de cette étude était d’établir l’étendue de la participation communautaire et les éléments qui empêchent et facilitent la participation des patients ayant souffert d**’**accident vasculaire cérébral après avoir quitté l'hôpital.

**Méthode:**

Cette étude fait partie d'une étude plus conséquente qui se concentre sur l'impact de l’éducation des aidants sur les personnes ayant survécu à un accident vasculaire cérébral et leur carrière. Cette étude longitudinale portait sur 200 patients ayant subi pour la première fois un accident vasculaire cérébral ischémique. Bien que les patients aient bénéficié d**’**un suivi à domicile 3, 6 et 12 mois après leur accident, cette étude se concentre sur les résultats de la participation au suivi à 12 mois après l**’**accident vasculaire cérébral. La capacité fonctionnelle des patients a été mesurée en utilisant l'indice de (IB) et l'indice de mobilité de Rivermead (IMR), et la participation a été mesurée en utilisant la liste de contrôle de la Classification Internationale du Fonctionnement, du Handicap et de la Santé (CIF). Des statistiques descriptives ont été utilisées par analyser les données.

**Résultats:**

Les patients ont connu des difficultés sévères à complètes pour entreprendre des tâches uniques et multiples sans aide 12 mois après avoir quitté l'hôpital. Ils avaient des difficultés à préparer les repas, à s**’**acquitter des tâches ménagères et dans leurs interactions avec d**’**autres personnes, et ils avaient des difficultés avec la vie communautaire et la participation aux activités récréatives et de loisirs. La famille proche et les attitudes de la société sont considérées comme des facilitateurs à la participation communautaire alors que les amis, les services de transport et services de sécurité sociale sont considérés comme des barrières à la participation communautaire.

**Conclusion:**

La capacité du patient à socialiser et à participer à la vie communautaire est faible à l'heure actuelle. Il est nécessaire de s'attaquer aux barrières identifiées à la participation communautaire afin d'améliorer la participation des patients à la communauté après un accident vasculaire cérébral.

## Introduction

### Setting

The study was focused on first-time patients with ischaemic stroke who were admitted to hospital in Johannesburg, South Africa.

### Key focus

After a stroke, the attainment of independent community ambulation is a challenging rehabilitation goal.^1^ If patients do not have an adequate ambulatory ability, their ability to participate in the community is affected directly.^2^ About 66% of stroke patients who are in the community need help with at least one activity of daily living.^3^ The need to understand the level of community participation and factors which either facilitate or inhibit that participation, cannot be overemphasised.

### Background

Improvement in health-related quality of life (HRQL) defines the main goal of rehabilitation.^4^ The three main components or domains of HRQL that are focused on post-stroke are, physical function, mental health, and participation. The World Health Organisation (WHO) describes participation as ‘involvement in life situations’.^5^ They elaborate by stating that the definition of participation brings in the concept of involvement. This involvement (participation) is affected by environmental and personal factors. Consequently, the measurement of activities of daily living only in this subgroup becomes inadequate to reveal the full extent of the impact of stroke according to the International Classification of Functioning, Disability and Health (ICF) model. These individuals may still have limitations in physical functioning, instrumental activities of daily living and participation^6^ and thus will rely on caregivers.

### Trends

Patients with stroke generally function better in activities relating to daily living than they do in social activities or interactions.^7^ Stroke patients living with another adult (a caregiver), however, demonstrate a lower degree of functioning in activities of daily living, but have better community participation.^7^ The adult carer will perform most of the activities of daily living for the patient and thus will not give them an opportunity to practise. Community participation will improve, because the adult carer is able to assist the patient with transfers and moving from one location to another.

Accessibility of community facilities is found to be one of the predictors of social integration of patients with stroke.^8^ Thus, if the facilities are not accessible, it becomes less likely that the patient will integrate into the community. This also affects compliance with medication, as indicated by Hale et al.,^9^ who established that medication non-compliance is largely because of financial and transportation difficulties in attending clinics. Inability to participate in the community could be ascribed to physical complaints such as pain in the joints post-stroke.^9, 10^ Equally important is the role of environmental factors in determining the extent to which an individual will be able to participate in community activities post-stroke.^11^

Poorly functioning patients result in an increased caregiving burden, in agreement with Ilse et al.^12^ who state that the patients’ functional and activity level play an important role in predicting caregiver strain during the sub-acute phase, whereas the participation level becomes more important over time. This emphasises the importance of assessing the participatory level of post-stroke patients if one is to have a complete picture of a patient's post-stroke improvement and the associated caregiving burden.

### Objectives

The aim of this study was to establish the level of community participation of patients at 12 months post-stroke and the associated factors impacting on that participation.

### Contribution to field

Most of the research that is available on post-stroke patients focuses on inpatient rehabilitation and outcomes. The need for us to understand the success of any post-stroke interventions cannot be overemphasised and hence the importance for us of gaining some insight into community participation of patients post-stroke. This understanding will give an indication of how effective the post-stroke interventions really are and will identify areas that need more strengthening to improve patient quality of life.

## Ethical considerations

The study obtained ethical clearance from the University of the Witwatersrand Human Research Ethics Committee (Clearance Number M050328).

### Potential benefits and hazards

There were no potential hazards to the participants. The participants were assured that the study would not interfere with their rehabilitation or treatment in any way.

### Recruitment procedures

Potential patients for the study were identified in the wards. The study procedure was then explained to them, as well as an explanation as to what consenting to take part in the study meant. They were only included in the study if there was consent.

### Informed consent

The purpose of the study was explained to the participants and they were told that participation was on a voluntary basis.

### Data protection

Data that were collected from the study were kept under lock and key and were only used for the purpose of the study.

## Methods

Data were collected by using a demographic questionnaire as well as the ICF (Part 2: Activity Limitations & Participation Restriction, and Part 3: Environmental Factors). Patient functional ability was established by using the Barthel Index (BI) and the Rivermead Mobility Index (RMI).

The RMI was developed from the Rivermead Motor Assessment by Collen et al.^13^ The focus of this instrument is on body mobility. The RMI comprises 14 questions and one direct observation. The RMI covers a range of activities that assess how mobile the patient is, ranging from bed mobility to running. If the patient is unable to perform the aspect of mobility they score a ‘0’; if they are able to perform it independently they score ‘1’ and the values are added. This allows for a total out of 15. If they score 15 per 15, they are deemed to be completely functional as far as their mobility is concerned. The RMI was shown to be a valid tool for assessing mobility in patients with stroke.^14^ It was also shown to be reliable to a limit of 2 points out of 15.^13^ A coefficient of reproducibility of greater than 0.9 was also established,^15^ whereas Green et al.^16^ found a mean difference and reliability coefficient of 0.3 ± 2.2 confirming that the RMI is a valid and responsive instrument when measuring mobility in patients with stroke.

The BI was used to gather information on the patient's functional independence in activities of daily living. The 10-item BI was published by Mahoney and Barthel^17^ to measure functional independence specifically directed at the personal and domestic activities of daily living. It comprises 10 questions, which address bowel and bladder management, grooming, toilet use, feeding, transfers, mobility, bathing and dressing. The values assigned to each item are based on the time and amount of actual physical assistance required if a patient is unable to perform the activity.^17^ The total score for the original BI was 100 and the higher the score, the better the functional ability of the patient. The original BI as put forward by Mahoney and Barthel^17^ has since been modified substantially. The BI developed by Collin and his colleagues,^18^ for example, has a maximum score of 20. This BI variation (the Collin 20-point) has been shown to be completely valid, reliable, appropriate and clinically significant.^18^ This version was used in this study.

In a study investigating the test-retest reliability of the BI, Green et al.^16^ found that the mean difference between testing was only 0.4 and a reliability coefficient of 2.0 was found, indicating good reliability with little bias. The study of Salter et al.^19^ showed that the BI had good responsiveness with a noteworthy ceiling effect of only 27% seen post-discharge from rehabilitation facilities. Their study also found the BI to have excellent test-retest (regardless of the skill of the evaluator) and inter-observer reliability, as well as excellent internal consistency.

The ICF, Disability and Health (ICF) checklist provides a standard language and a universal and globally accepted framework and classification that comprehensively address human experiences in relation to functioning and health. The ICF was endorsed by the World Health Organisation (WHO) in 2001 as a modification or improvement of the International Classification of Impairments, Disabilities, and Handicaps (ICIDH).^20^

The ICF allows one to measure how an individual fares when the capacity to carry out an activity and actual performance is involved. Capacity refers to what a person can do under the best circumstances and performance refers to what the person can actually do in day-to-day life.^21^ The ICF has positive as well as negative terms to reflect both capacity and difficulty.^22^ The positive terms are body structure and function, activity, and participation, whereas the negative terms are impairments, activity limitations and participation restrictions. The validation process of the ICF is an ongoing development in which all the evidence gathered during its implementation will be integrated.^23^ The fact that the ICF was borne out of a worldwide comprehensive consensus process over several years arguably gives it a degree of validity. Other studies have found the ICF to have exhaustiveness or width because it was shown to be able to cover all aspects of the patient experience.^24, 25^

### Setting

This study formed part of a larger study that was assessing the impact of caregiver training on stroke survivors and their caregivers. The study comprised 200 patients with first-time ischaemic stroke. Stroke survivors from Chris Hani Baragwanath Academic Hospital (CHBH) participated in the study. The hospital caters mainly for the surrounding, largely black, population of Soweto in Johannesburg, South Africa.

### Design

The focus of this report is on community participation at 12-months post-discharge from hospital and the factors that influence this participation. Patients were followed up and assessed at home post-discharge.

### Procedure

In order to attain the required sample size for the study, all consecutive patients with first-time ischaemic stroke fitting the inclusion criteria were approached by either the researcher or the research assistant for their permission and initial screening for inclusion into the study until the sample size was reached.

The BI, the RMI and the ICF were administered to the patients before discharge, and at 3-months, 6-months and 12-months follow-up at home. Administration of the instruments was carried out by the researcher. It should, however, be noted that the focus of the data presented in this paper is on the 12-months follow-up. In the event of the patient having speech problems and unable to provide some of the information required for the data collection process, the caregiver was asked to provide the information.^26^ During the administration of the instruments, any other information that was given by the patient or the caregiver which was deemed relevant, was recorded. Data collection was carried out from 2006 to 2010.

### Analysing

Descriptive statistics were used to analyse the data for the demographic information and level of community participation of the patients. The means and standard deviations of the various data categories were calculated as was appropriate for the demographic variables of the patients and the caregivers, for example, for age and for the RMI and BI scores. The data obtained from the BI and RMI provided a degree of functional independence or dependence of the stroke survivor.

## Results

### Demographics of the study sample

At the beginning of the study, there were 87 (43.5%) male and 113 (56.5%) female patients in the study sample. The mean age of the male patients was 52.1 ± 11.4 years whereas it was 54.1 ± 11.4 years for the female patients. A large percentage (71%) of the patients (142) was unemployed and a large percentage of them (49%) was single. A large percentage of the patients (60%) was cared for by relatives, followed by 39% who were cared for by spouses. At the 12-months follow-up, there were 114 patients. Thirty-eight per cent of the patients died whereas 10 were lost to follow-up.

### The ability of the stroke survivor to socialise and participate in community issues

A summary of the patients’ ability to socialise and participate in the community at 12 months post-discharge is provided, including the environmental factors that were barriers or facilitators.

### Extent of general participation restriction

For this section, results that showed significant findings are highlighted. According to the ICF, *the performance qualifier* assesses the patient's current ability to perform activities whereas *the capacity qualifier* assesses the patient's ability to carry out activities without any form of assistance. It is therefore expected that in some cases it may be more difficult for patients to perform activities without assistance.

**FIGURE 1:**
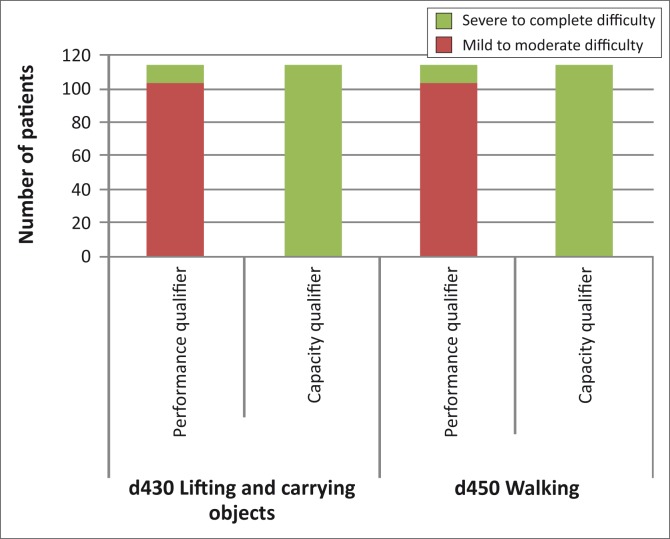
The extent of mobility performance and activity limitation for the patients.

No participants (100%) could carry out single and multiple tasks without assistance at 12 months post-discharge from hospital (Table 1). All the participants indicated an inability to lift and carry objects and walk without assistance (capacity) at 12 months post-discharge (Figure 1). None of the participants was able to carry out domestic activities without any difficulty at 12 months post-discharge from hospital (Table 2). All the participants had mild to moderate and severe to complete difficulty in basic interpersonal interactions and formal relationships at 12 months post-discharge (Table 2). At 12 months, 26.5% of the control group and 24.6% of the experimental group still had severe to complete difficulty with community life whereas all the participants (100%) had mild to moderate difficulty with recreation and leisure activities (Table 2).

**TABLE 1:** The extent of general participation restriction for the patients (*n* = 114).

Tasks	Level of difficulty	Performance Qualifier		Capacity Qualifier	
	
		*n*	%	*n*	%
**Undertaking single tasks**					
	No difficulty	0	0	0	0
	Mild to Moderate difficulty	53	46.5	0	0
	Severe to Complete difficulty	61	53.5	114	100
**Undertaking multiple tasks**					
	No difficulty	0	0	0	0
	Mild to Moderate difficulty	53	46.5	0	0
	Severe to Complete difficulty	61	53.5	114	100

*n*, Given as number of patients.

**TABLE 2:** The extent of general participation restriction in domestic activities for the patients (*n* = 114).

Tasks	Level of difficulty	Performance Qualifier		Capacity Qualifier	
	
		*n*	%	*n*	%
**Activities**					
Preparation of meals	No difficulty	0	0	0	0
	Mild to Moderate difficulty	114	100	0	0
	Severe to Complete difficulty	0	0	49	100
Doing housework	No difficulty	0	0	0	0
	Mild to Moderate difficulty	114	100	0	0
	Severe to Complete difficulty	0	0	114	100
**Personal relationships and interactions participation and limitation of patients**					
Basic interpersonal interactions	No difficulty	0	0	0	0
	Mild to Moderate difficulty	55	49.1	0	0
	Severe to Complete difficulty	26	49.1	114	100
Formal relationships	No difficulty	0	0	0	0
	Mild to Moderate difficulty	114	100	0	0
	Severe to Complete difficulty	0	0	114	100
**Activity participation and limitation in community, social and civic life**					
Community Life	No difficulty	0	0	0	0
	Mild to Moderate difficulty	85	74.6	0	0
	Severe to Complete difficulty	29	22.8	114	100
Recreation and leisure	No difficulty	0	0	0	0
	Mild to Moderate difficulty	114	100	0	0
	Severe to Complete difficulty	0	0	114	100
Political life and citizenship	No difficulty	0	0	0	0
	Mild to Moderate difficulty	67	58.8	0	0
	Severe to Complete difficulty	47	41.2	114	100

*n*, Given as number of patients.

The extent of general participation restriction for the patients has been outlined (Table 1).

## The environmental factors that influenced the patient-ability to function in the community

**FIGURE 2:**
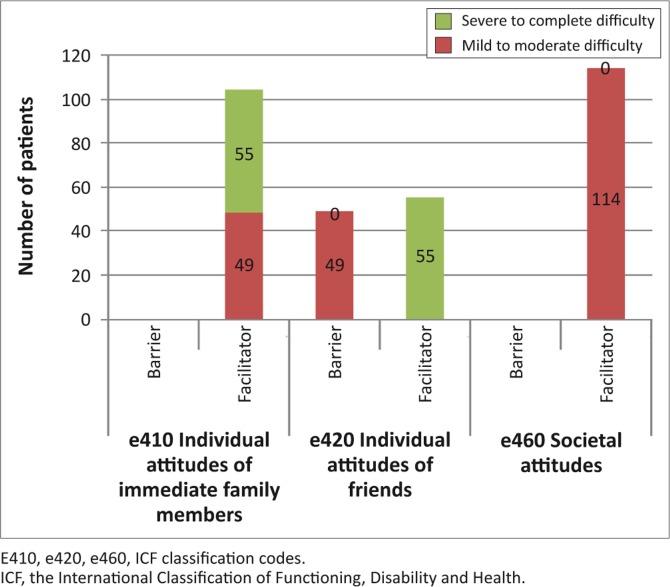
The attitudes environmental factors that influenced the patients’ ability to function in the community (*n* = 114).

**FIGURE 3:**
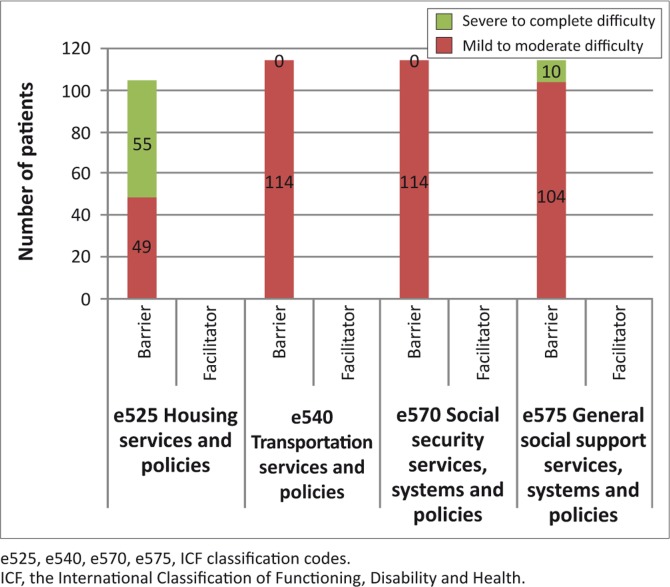
The services, systems and policies factors that influenced the patient-ability to function in the community (*n* = 114).

The support of the immediate family and that of personal care providers and assistants was seen as a facilitator to activity participation. More than 50% of the patients saw the support of friends as barriers. Acquaintances, peers, colleagues, neighbours and community members were seen largely as facilitators to activity participation (Table 3). The individual attitudes of the immediate family members were viewed as facilitators, whereas that of society was seen as facilitative (Figure 2). All the participants viewed the social security services and systems and policies as mild to moderate barriers, and the general social security services and systems and policies as mild to complete barriers to their extent of participation in the community. Housing policies were also considered as a mild to complete barrier (Figure 3).

**TABLE 3:** The support and relationships environmental factors that influenced the patient-ability to function in the community (*n* = 114).

Variables	Level of difficulty	Barrier		Facilitator	
	
		*n*	%	*n*	%
**Immediate family**					
	No barrier and facilitator	0	0	0	0
	Mild to Moderate barrier and facilitator	0	0	59	51.7
	Severe to Complete barrier and facilitator	0	0	55	48.2
**Friends**					
	No barrier and facilitator	0	0	0	0
	Mild to Moderate barrier and facilitator	65	57.0	49	43
	Severe to Complete barrier and facilitator	0	0	0	0
**Acquaintances, peers, colleagues, neighbours and community members**					
	No barrier and facilitator	0	0	0	0
	Mild to Moderate barrier and facilitator	49	43.0	10	8.8
	Severe to Complete barrier and facilitator	0	0	55	48.2
**Personal care providers and personal assistants**					
	No barrier and facilitator	0	0	0	0
	Mild to Moderate barrier and facilitator	0	0	49	43.0
	Severe to Complete barrier and facilitator	0	0	65	57.0

*n*, Given as number of patients.

The mean distribution of the BI and RMI scores at 12 months have been tabulated (Table 4).

**TABLE 4:** Mean Barthel Index and Rivermead Mobility Index scores at 12 months.

Functional Measure	Mean	s.d.
Barthel Index	13.0	2.2
Rivermead Mobility Index	8.4	3

s.d., standard deviation.

## Discussion

### Outline of the results

The aim of this study was to establish the level of community participation of patients with stroke at 12 months post-stroke and the associated factors impacting on that participation. The patients had problems with undertaking single and multiple tasks. At 12 months, 53% of the control group and 54% of the experimental group had severe to complete performance difficulty in undertaking multiple tasks, with 55% and 52% respectively having severe to complete performance difficulty with undertaking single tasks. All the patients were dependent on their caregivers for single and multiple tasks as shown by all patients not being able to carry out any tasks without help.

The patient-dependency on outside help can be explained by the poor functional levels that the patients exhibited at the 12 months follow-up. A score of 60% on the BI is the cut off between independence and more marked dependence, 40% or below indicates severe dependence and 20% or below reflects total dependence.^27, 28^ The mean BI score at 12 months for the patients was 13 ± 2.2 which was on average low. Low BI scores negatively affect patients’ ability to perform activities and participate in the community.^29^ The dependence they had, stemmed from their poor physical condition as depicted by the low BI and RMI scores.

In agreement with the poor BI and RMI scores, the patients had mild to moderate and severe to complete difficulty with the lifting and carrying of objects, and walking. The dependence of the patients on their caregivers is reinforced by the fact that they had severe to complete difficulty in carrying out the above-mentioned activities without assistance (capacity). Low physically functioning patients have role limitations and are very limited in their social functioning.^30^ As stated by Lord et al.,^1^ independent community ambulation is a challenging goal. It appears as if the same trends observed by Disler et al.^31^ persist today. In their study, they found that stroke was the largest cause of disability (23.7%) and that the majority of the patients with disability that they saw had problems with locomotion. Hale et al.^9^ also found that patients in Soweto, South Africa, struggled with gait whilst in the community and recommend that safe walking must be ensured before discharge. A systematic review by van de Port et al.^32^ showed that gait-oriented training interventions have a significantly positive effect on both gait speed and walking distance.

All the patients had mild to moderate difficulty in preparing meals and doing housework without assistance at the 12-months follow-up period, suggesting that caregiver education might have contributed positively towards their mobility (as needed for these activities) in the community. It should, however, be noted that the ICF data were collected mainly from patient interviews and as such they may have overestimated their ability to perform mobility activities. It was demonstrated as such when post-stroke patients could not make a simple return trip to the shops despite reporting that they were able to do so.^2^ Patient community participation could be higher mainly because of the help they receive from the caregivers.^7^ This seemed to have been the case in this study if one considers that all the patients in the study sample had severe to complete difficulty in carrying out the mobility subcomponents under discussion, when undertaken without help from their personal caregivers and assistants.

All the patients demonstrated some degree of difficulty with basic interpersonal and formal relationships. The majority of the patients expressed mild to moderate difficulty for the performance qualifier, and complete dependence on the caregivers as demonstrated by their severe to moderate difficulty for the same activities when without assistance (capacity qualifier). The high number of patients experiencing problems with personal interactions and relationships agrees with the finding by Hommel et al.^33^ who established that 78% of their study sample complained of social dysfunctionality despite having good functional abilities. The patients in this cohort did not have good functional abilities; they were very low-functioning and this could possibly explain the reported inability to socialise.

Patients struggled with community, social and civic life activity participation. They displayed mild to moderate and severe to complete difficulty with community life, recreation and leisure ability and political life. The patients again showed complete dependence on their caregivers, for all of them (100%) had severe to complete difficulty with the same activities when they were without assistance. This again agrees with the limited improvements that were noted in patients’ functional abilities at the 12months follow-up. Similar problems with socialisation were established in a previous study in the same geographical location with similar patients.^9^ Hale et al.^9^ reported that post-stroke patients received very few visitors and only two could manage to visit their neighbours. Higher social participation is associated with better physical function and vitality in post-stroke patients.^34^

The patients viewed the immediate family and personal care providers and assistants largely as facilitators. This agrees with the finding that the patients were largely dependent in activities of daily living and demonstrated severe to complete difficulty in carrying out activities without help. Stroke survivors receive help from their caregivers regardless of their functional abilities.^35^ Of particular interest was the percentages of those who thought that the immediate family were severe to complete facilitators (48.2%) at 12 months. This could be a consequence of one of two things; firstly, it could be a signal that the patients appreciated the role of the caregivers more as time passed, or secondly, it could signal an increased dependency on the caregivers, which would be a perturbing sign. One would expect that, as patients regain some of their functional abilities, they would rely less on the caregivers for carrying out activities of daily living. One needs to take note, however, that although the patients’ functional abilities generally improved over time; they did not do so to satisfactory levels at the end of the 12 months of the study.

Patients regarded their friends as being mild to moderate barriers (57) to their ability to participate in the community. One of the consequences of stroke is a limitation of social participation.^36^ The major concern for the patients seemed to have been the fact that they could no longer ‘hang out’ with their friends as they used to do before the stroke and the visits from the friends had diminished. To quote one patient:

‘Ever since I came back from the hospital, my friends have hardly been here to spend some time with me, it is as if I have stopped existing for them and that pains me a lot.’ [Participant 1, Male, 58 years old]

One can explain this through the limited functional abilities that the patients exhibited. Going out with them (the patients) would have meant a lot of physical work for the friends and so they opted out. One can only speculate that if the patients were more functional, their friends would have found it much easier to spend time with them.

The above scenario is strengthened further by the finding that at 12 months, 43% of the patients thought that their friends’ attitudes were mild to moderate barriers to their ability to function in the community. They were, however, happy with the attitudes of the immediate family members, the personal providers and society care. The patients found the general population to be helpful, as one patient put it, ‘The public is very understanding; when they see me coming, they either offer to help or they give way which helps with my mobility to some extent.’ However, the same could not be said about their friends. This is supported by the finding that 48.2% of the patients perceived acquaintances, peers, colleagues, neighbours and community members as facilitators to community participation.

One major source of concern for the patients was the availability of housing and accessibility of social grants (the disability grant to be exact). All participants considered the housing services and policies to be either mild to moderate or severe to complete barriers. Poor housing conditions and environmental factors, poverty and its deep effects on body and spirit, poor education and low literacy are greater causes of poor health than racially biased medical care.^37^ The patients wished to have access to better housing and to quote one patient:

‘The government needs to give priority to people with disabilities when it comes to housing. If I was staying in a better house, I am quite sure I would be able to participate in the community more.’ [Participant 2, Female, 64 years old]

The issue of social grants was also problematic for most patients who are from a low socio-economic level. People of low socio-economic status have worse health and are most likely to receive a disability pension.^38^ Patients complained that they did not have the means to go to the social welfare offices to make the necessary grant applications, whereas in a few cases they were not even aware that they qualified for disability grants. This cohort of patients was not employed and consequently would have benefited from social grants. As stated by Bonita and Beaglehole^39^ in an editorial, ‘stroke is a cause of poverty and is caused by poverty’. Patients who are able to go back to meaningful employment post-stroke report better health-related quality of life.^40^ The inaccessibility of social grants by persons with stroke also points towards coordination problems with the rehabilitation team. It questions the strength of the hospital interdepartmental referral system, especially between the ward, physiotherapists, occupational therapists, speech therapists and the social workers, the latter being responsible for processing the applications. As stated by Lincoln,^41^ clearly, coordinated rehabilitation is lacking.

It was worrying to note that 100% of the patients viewed transport services, systems and policies as mild to moderate barriers. This stemmed mainly from the fact that, if patients were using a wheelchair for mobility, they would then be asked to pay for taxi fare for themselves as well as for the wheelchair. In some cases they were not allowed onto the taxi and that impaired their ability to move around severely. As one patient put it:

‘being in a wheelchair is like a curse, you are being punished for being disabled, the taxi either does not stop for you or if it does, then you have to pay for yourself, the person helping you and the wheelchair making the whole business of moving around pretty expensive.’ [Participant 3, Male, 65 years old]

It is therefore quite clear that the transport and financial problems that patients have when in the community are major sources of limitation to community participation. Transportation problems amongst patients with stroke were raised in an earlier study^9^ and they appear to remain a major concern. This limits patients’ ability to move around and may even have contributed towards their inability to attend outpatient rehabilitation.

### Practical implications

The patient-ability to socialise and participate in community issues is currently poor. This is mainly affected by the patients’ poor levels of functional ability, which causes them to be dependent on caregivers for the execution of daily-living activities. Transport systems, services and policies, attitudes of friends and the design, construction and building products, and the technology for both public and private use were perceived as barriers to community participation and these should be addressed post-stroke.

### Limitations of the study

The use of a qualitative method of data collection might have shed more light on both the level of community participation post-stroke, and the barriers and facilitators to that participation. The findings from this study apply to a greater extent to populations of similar settings, which are low-resource settings.

### Recommendations

The provision of post-stroke rehabilitation should address and focus more on community participation.

The referral system between the discharging hospital and the local community health centre needs to be strengthened to ensure that all patients have access to rehabilitation post-discharge from hospital.

Interaction with social workers needs to be strengthened to ensure that disability-grant applications are made before the patient is discharged home, or the local social workers (in the community) need to be notified when a patient is discharged to their area.

## Conclusion

The patient-ability to socialise and participate in community issues is currently poor. Transport systems, services and policies, the attitudes of friends and the design, construction and building products and technology for both public and private use were perceived as barriers to community participation and an effort is needed to address these in order to improve patient-participation in the community post-stroke.
